# Terahertz Time-Domain Reconstruction of Coating Microstratigraphy on Gilded Surfaces

**DOI:** 10.3390/ma12233822

**Published:** 2019-11-21

**Authors:** Ilaria Cacciari, Daniele Ciofini, Hubert Baija, Salvatore Siano

**Affiliations:** 1 Istituto di Fisica Applicata “Nello Carrara”, Consiglio Nazionale delle Ricerche, v. M. del Piano 10, 50019 Sesto Fiorentino (FI), Italy; d.ciofini@ifac.cnr.it (D.C.); s.siano@ifac.cnr.it (S.S.); 2 Rijksmuseum Amsterdam, Museumstraat 1, Postbus 74888, 1070 DN Amsterdam, The Netherlands; h.baija@xs4all.nl

**Keywords:** THz Imaging, Gaussian fit, pulse-echo temporal profile, microstratigraphy, patination, overpaint, brass paint, gilding, gold leaf

## Abstract

Here, a systematic study in order to assess the potential of THz time domain reflectometry for measuring the thicknesses of overpaint layers applied on original gilded surfaces was carried out. The work is part of a thorough characterization campaign, which is going on at the Rijksmuseum for addressing the conservation problems of a set of 19th century gilded picture frames on which heavy coatings were applied in previous undocumented restoration interventions. To perform such non-invasive thickness measurements, an analytical protocol based on Gaussian fits of the THz pulse-echo temporal profiles was optimized through the preparation of suitable technical samples and the comparison with direct thickness measurements. Finally, the methodology was validated by characterizing the microstratigraphy of an original sculptural element from a gilded picture frame in the Rijksmuseum collection. The results achieved show the effectiveness of the present approach in revealing multi-layered dielectric microstructures with a spatial resolution of about 30 µm when using a spectral range up to 1.5 THz.

## 1. Introduction

Terahertz (THz) technology has been shown to be increasingly useful in a plethora of practical applications ranging from biosensing [1], to imaging [2], as well as for wireless communications [3]. One of the most intriguing features of THz waves is that they are well suited for non-destructive characterization of multi-layered dielectric materials [4]. This has been successfully exploited in the food industry [5,6,7], medicine [8,9,10,11], and security [12,13,14], as well as in cultural heritage, for investigating artworks [15,16,17,18]. In time domain, a THz spectroscopy setup, both in transmission and in reflection, can be used as an excellent tool for non-destructive measurement of dielectric thicknesses. In this case, the application fields may cover pharmaceutical coating inspections [19,20], automotive-paint quality check-ups [21,22], and cultural heritage [23,24]. In these cases, the possibility to perform non-contact measurements represents an appealing feature. Besides THz time of flight, existing non-destructive techniques for direct thickness measurement may include ultrasound and photoacoustic probes, ellipsometry, optical coherence tomography (OCT), NIR confocal laser scanning microscopy. Within such an analytical set, the THz approach extends the potential of optical techniques, which can be used only on sufficiently transparent layers, and allows avoiding the strict mechanical contact needed for pressure wave probing. Hence, the THz probing can be used as a valid complementary technique in micro-stratigraphic characterization of multi-layered dielectric materials in transmission or reflection configuration.

In principle, with THz waves, the thickness of each layer in a stratified sample can be calculated from the measured delay of the reflected echoes and the propagation constants of the media. In the frequency domain, the Fourier deconvolution can be used in order to extract an impulse response from the measured echo pulses and to estimate the propagation constants [25,26]. This approach can be used for thicknesses above the half of the coherence length of the THz pulse, and therefore is unsuitable for thin layers analysis.

Moreover, when thin layers constitute a multi-layer system, the reflected echoes in a THz time-of-flight setup are temporally overlapped. Suitable and often complex mathematical models are hence required to analyze measured signals. An advanced regression procedure based on the Debye model has been proposed, which involves Fourier analysis combined with genetic optimization algorithm [22].

To decrease the minimum thickness measurable with time-of-flight reflection measurements, a numerical fitting procedure based on multiple regression algorithms has been proposed [21]. In particular, the signal reflected from a metal surface has been measured and used to fit the one reflected from a dielectric layer placed in the same position. The efficiency of this method has been proven only on a single layer and by neglecting the effect of dispersion in the refractive index. In this work, we aim at improving this approach, by considering a multi-layered system and by taking into account the THz echo pulse distortion.

The proposed method has been exploited for studying the microstratigraphic sequences encountered on a set of 19th-century gilded picture frames on which patinations were applied in undocumented restoration interventions in order to reveal their golden appearance. Thus, besides original preparation and gold leaf relics, overlapped re-preparation and a finishing layer of a few tens of microns were found on the mentioned frames, which represent interesting cases of thin multi-layered material systems. In particular, the aim of the present work was to study whether THz radiation can penetrate through such coats that were applied upon the original surfaces and provide information on the whole stratigraphy. Non-invasive imaging techniques capable of mapping buried remnants of gilding could be greatly supportive in view of planning possible conservation treatments aimed at removing late gold-like patinations, which disguises the original surface textures.

Here, multi-layered paint mock-ups simulating the stratigraphic sequences of the picture frames were prepared and measured with THz imaging, in order to define and test the analytical procedure. The latter has then been successfully exploited to the study of the complex stratigraphy of an original frame ornament.

## 2. Materials and Methods

### 2.1. Multi-Layered Samples and Original Frame Ornament

**Figure 1:**
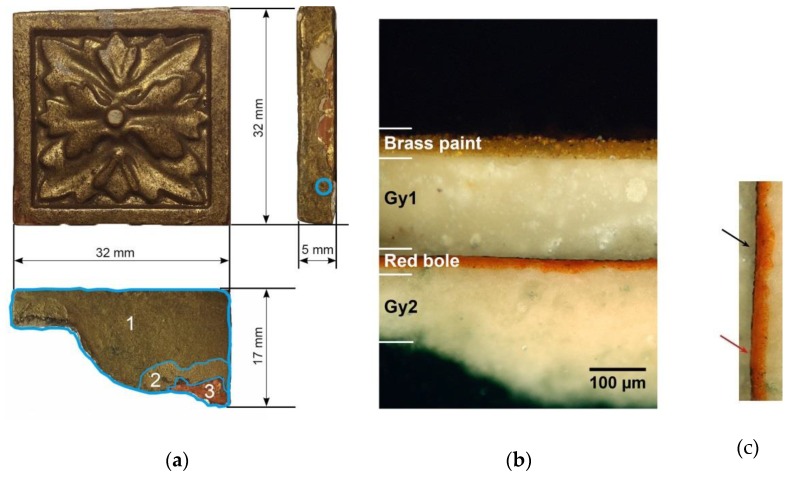
(**a**) Original frame ornament; (**b**) cross-section microphotograph (bright field with crossed Nicols) of the circled area in Figure (**a**); (**c**) detail of the red bole and gold leaf interface of Figure (**b**): the gold leaf (black arrow) in some points is completely lost (red arrow).

Multi-layered samples were prepared in order to simulate the stratigraphy of a cast ornament, namely a fragment belonging to a gilded frame in the Rijksmuseum collection (Figure 1a). In order to study its layers build-up, a small area (circle in Figure 1a) has been sampled in order to prepare a cross-section.

This has been micro-photographed under dark-field illumination using a Nikon Eclipse L150 optical microscope (obj: 20×) (Tokyo, Japan) equipped with a Nikon D80 DSLR camera (Tokyo, Japan). As displayed in the cross-section image in Figure 1b, the four main layers, labeled from Gy1 to brass paint, can be recognized. The substrate (Gy2) is made up of compo (i.e., composition), a dense mixture of calcium carbonate/sulfate and protein glue.

During the 19th century, compo was usually preferred to carved wood, as it is much faster to produce and more stable as a substrate for gilding [27]. In the sampling area, the compo substrate is followed by a 20–30 µm thick colored bole layer, which may vary greatly from point to point of the sample, as well as by gold leaf (less than 1 μm thick), a burnished water gilding.

Traces of the latter may be appreciated in the external perimeter of the ornament, mainly where the over-paint is missing (Figure 1a).

Indeed, the loss of the original gilding has definitely motivated the painting operation of the whole ornament. This was carried out by applying a *gesso* layer (Gy1) on top of the residual original gilding that represents the ground of the outmost finishing layer. The latter was realized first by sealing the underlying *gesso* preparation with an oil-resin topcoat, and then by finishing it with an organic-binder paint layer with a significant brass powder load.

THz measurements were performed on mock-ups prepared in order to simulate the stratigraphy of original 19th century gilded picture frames. To reproduce it, ground layers (Gy1 and Gy2) were both realized by blending together *gesso sottile* and a protein glue size (2:1 pigment to binder ratio), in accordance with the treatise of Cennini [28].

**Figure 2:**
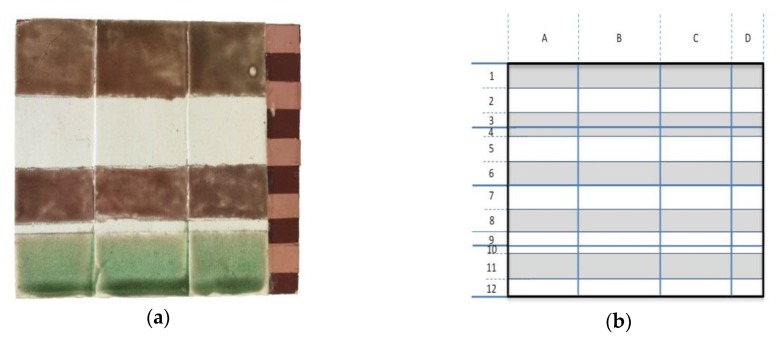
(**a**) Multi-layered sample (75 × 52 mm); (**b**) schematization of the different areas analyzed. In grey are indicated the areas containing the metal layer (copper tape). Lines 1, 2, 3 are varnished with Colophony (Col-Eth); lines 4, 5, 6, 10 are not varnished; lines 7, 8 are varnished with Shellac (She-Eth); lines 10, 11, 12 are varnished with Sandrac (Oil-San); Column A contains a very fine gypsum layer (Gy1); column B has a thick Gy1 layer; and column C contains the thickest Gy1 layer.

Moreover, considering that the thickness of the compo substrate may reasonably be overlooked, as too thick for THz radiation to emerge and be detected, no restrictive precautions were taken for executing the Gy2 layer. It was applied to a wooden panel (50 × 70 mm sizes) having a thickness of 400–500 µm. Red bole in a protein glue medium (1:1 p/b by weight) was then spread out with a brush. Once dried, five strips (5 × 60 mm) of copper adhesive tape (60 µm thick) representing a gilding finish were stuck onto the bole-coated surface. The prepared sample and its layout are displayed in Figure 2a,b.

To account for the high thickness variability that ground layers can have in the actual frames, three gesso layers (Gy1) of different thicknesses were superimposed to the bole/metal surface. Last, to reproduce the imitation gold topcoat, homemade brass paint varnishes were formulated using a commercial copper-zinc powder marketed by CTS Europe (Altavilla Vicentina, Italy). Varnish formulations were achieved according to a known methodology [29].

In brief, shellac (She-Eth) and colophony (Col-Eth) resins were both dissolved in ethanol (30% *w*/*v*), while the oil-sandarac mixture (Oil-San) was softened up to 150 °C and then heated up to 285 °C. Once the temperature dropped down to 60 °C, rectified turpentine oil was added in a 1:1 ratio (wt:wt).

In total, 0.6% of the weight of brass powder was added to the as-prepared varnishes, gently stirred by hand with a soft brush, and then applied to the three differently thick gesso layers, as illustrated in Figure 2a. Here, as follows the brass paints are named using only the names of the resins and oil.

### 2.2. THz Imaging Setup

The THz time domain spectrometer used for imaging measurements has been mounted in reflection geometry [30,31]. In order to create THz pulses, the ultrafast pulses (70 fs) from a fiber laser emitting at 1550 nm (Toptica, Femto-FErb 1560, Gräfelfing, Germany) have been split into two arms: one for detection and the other for generation. A series of mirrors and lenses have been used to guide the ultrafast pulses to the fibers coupled with the emitter photoconductive antennas (PCA).

The emitted THz pulse has a bandwidth extending from 0.01 to 1.5 THz. It has been focused with a Teflon lens on the sample (working distance 30 mm) at an incident angle of 30° with respect to the normal direction. The reflected signals are focused back using a second plastic lens on the detector antenna at 30°. The temporal delay between the THz pulse and the ultrafast pulse on the detector can be adjusted by introducing an optical delay line motorized stage in the detection arm. By varying the optical path length, the THz time domain can be sampled, thus producing both amplitude and phase information.

### 2.3. Fitting Procedure for Thickness Estimation

**Figure 3:**
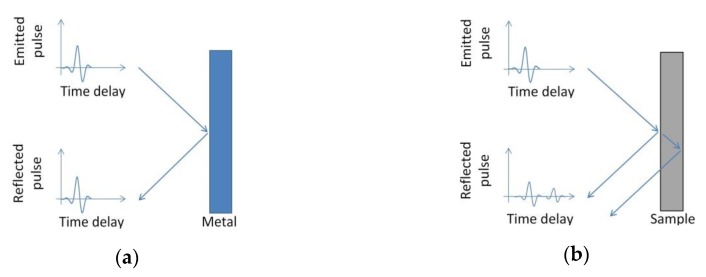
(**a**) The THz pulse emitted by the PCA is focused on a metal plane surface, the reflected pulse is considered equivalent to the emitted one; (**b**) reflection from a sample placed in the same position of the metal surface, the reflected signal comprises two pulses originating from the reflections at air/sample and sample/air interfaces.

The THz pulse emitted has been focused onto a metal plane surface; the reflected signal (reference) is sketched in Figure 3a. In general, this pulse could be enhanced with a careful alignment of the reference surface, in addition to the two antennas. Once this optimization has been performed, the pulse reflected from the metal surface can be measured and considered as the reference signal.

When a mono-layered sample is placed in the same position of the metal surface, the reflected pulse comprises two pulses originating from the reflections at air/sample and sample/air interfaces (Figure 3b). The temporal shape of any reflected pulse becomes more complex whenever a multilayer sample is considered.

Here, we propose to simplify the description of the temporal shape of any reflected THz pulse by using a nonlinear curve fitting algorithm. The main idea is that it is possible to fit any reference pulse (measured in time domain) with a linear combination of *n* Gaussians, and to use this result in order to study the reflected pulses from any multi-layered sample. This is motivated by the assumption that the THz electric field is modeled as proportional to the time derivative of the photo-current induced in the gap of the antenna [32].

Since the photo-current is assumed as Gaussian, we have assumed that its derivative can be approximated with a linear combination of Gaussians. A multi-peak fitting algorithm based on the Levenberg-Marquardt algorithm [33,34] has been considered in order to determine the set of parameters that best fit the entire reference pulse in time domain (*y_r_(t)*). The fitting model used is based on the sum of n Gaussians and an offset:

(1) $$y_{r}\left(t\right)=\sum_{1}^{n}a_{i}\left(e^{-\frac{{\left(t-m_{i}\right)}^{2}}{2s_{i}^{2}}}\right)+offset,$$

where *a_i_*, *m_i_*, *s_i_* are respectively the amplitude, the center, and the width of the *i*-th Gaussian. In general, several indicators are available to test the quality of the fitting results. Here, we have considered the weighted mean square error between the best nonlinear fit and the input data. This indicator is known as residue.

Once the reference pulse is fitted, all the parameters in Equation (1) are known and can be used to study the reflections from a multi-layered sample. The case of reflection from a sample with more than two interfaces is generally considered to be a complex case, since many effects, including Fabry-Perot resonances from inner interfaces, can occur. We propose in time-domain a simplified model in which the reference pulse plays a crucial role.

Let us consider the generic case of a THz pulse emitted by a PCA and focused on a mono layer sample. At the air/sample interface, the pulse is partially reflected to the detector and partially transmitted through the sample as is schematically depicted in Figure 3b. At the sample/air interface, the pulse is then reflected back to the detector. The reflection from a single-layered sample is hence constituted by two pulses.

When the sample is placed exactly in the same position as that of the reference metal mirror, in time domain, the first pulse shows a peak in the same position as that of the reference (Figure 3b).

Otherwise, a time delay is measured. Moreover, the effect of the reflection at the first interface of the sample can also be observed, also in attenuation and modification of the emitted pulse shape. This reflection can be roughly modeled as by considering an attenuation and time-shift of emitted pulse. In this simplified scheme, the emitted pulse is assumed equivalent to the reference pulse (Equation (1)). The first reflection can hence be expressed as follows:

(2) $$y_{1}\left(t\right)=A_{1}y_{r}\left(t+t_{1}\right),$$

where *A*_1_ represents the attenuation factor and t_1_ the time delay as compared to the reference pulse.

This representation takes into account a possible distortion of the incident pulse introduced due to dispersion effects. A similar description holds for the pulse reflected at the sample/air interface, which is delayed as compared to the first one (*t*_2_ *> t*_1_) since it travels through the sample, and significantly attenuated due to absorption material:

(3) $$y_{2}\left(t\right)=A_{2}y_{r}\left(t+t_{2}\right).$$

It should be observed that the independence of Equation (3) from the frequency represents an approximation, which holds in the limit of thicknesses much lower than the penetration depth of the THz radiation.

The sum of the two reflections expressed in Equations (1) and (2) represents what is effectively measured by the detector, apart from an offset factor that should be introduced in order to take into account the effect of the noise.

This scenario can be extended to multi-layered samples, in which there are *N* interfaces, and hence *N* reflections can contribute to the detected pulse:

(4) $$y_{N}\left(t\right)=\overset{N}{\underset{1}{\sum }}A_{i}y_{i}\left(t+t_{i}\right)+offset.$$

Unfortunately, in many practical applications, the stratigraphy of a multi-layered sample is not known, therefore the number of the interfaces cannot be easily established a priori. We have considered reflections up to 7 interfaces; further reflections give rise to pulses below the noise level and therefore have been ignored.

The measured signal reflected from a multi-layered sample is then fitted with the Levenberg-Marquardt algorithm in accordance with the simplified model of Equation (4).

The time delays obtained allowed for an estimation of the thickness of each layer in the sample, once providing the refractive index of the layer [35,36].

## 3. Results and Discussion

### 3.1. Cross-Section for Direct Thickness Measurement

For each area in the multi-layered sample (Figure 2), a cross-section has been prepared and five microphotographs analyzed under bright field illumination using a Nikon Eclipse L150 optical microscope (obj: 10×) equipped with a Nikon D80 DSLR camera.

**Figure 4:**
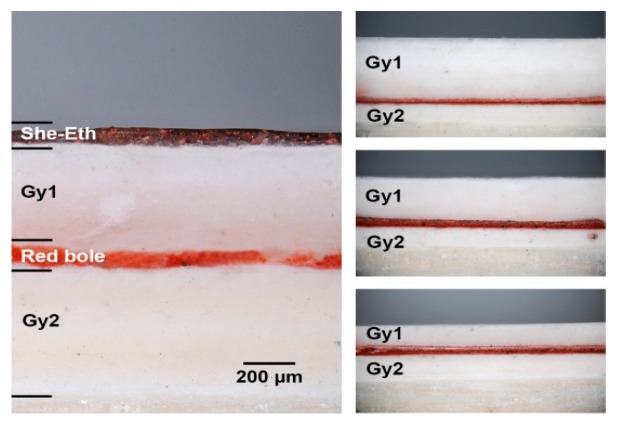
Cross-section microphotographs from (left) A7, (upper right) A5, (center right) B5, (lower right) C5 areas (see Figure 2).

In Figure 4, five exemplary micro-photographs of cross-sections prepared in different areas are reported. For each area, the resulting thickness measurements have been averaged and the maximum absolute difference was considered as the error. The results of the optical thickness measurements are reported in Table 1, Table 2, Table 3 and Table 4.

### 3.2. THz Thickness Measurement

As described above, a simplified modeling of the temporal shape of any reflected THz pulse is obtained by using a Gaussian fitting algorithm (Equation (4)). The number of most intense peaks in the reference pulse provides indication about the selection of the best number of Gaussians to be used in Equation (1). It has been observed that six Gaussians could provide the lowest residue. This means that the set of parameters to be determined is 19 (including the offset).

**Figure 5:**
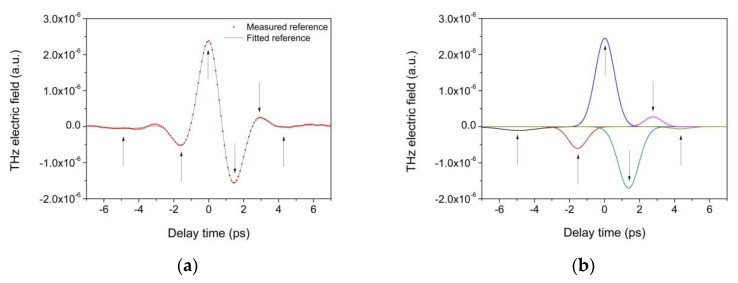
(**a**) Measured (scatter plot) and fitted (line plot) reference pulse; (**b**) Six Gaussians used in the fitting procedure.

In general, a fitting algorithm may converge to different local minima, depending on the values of the initial guesses, and as a result this may affect the reliability of the fitting. Since there is no sense in forcing any iterative algorithm to work too hard by initializing it with intentionally modest initial guesses, the correct practice is to define the initial guesses in accordance with an arbitrary criterion. We have observed that among the 19 fitting parameters, the six Gaussian centers heavily affect the fitting results. Therefore, their initial guesses have to be chosen with great care. In looking at the shapes of the reference pulses (scatter plot in Figure 5a), it is easy to identify the position of at least two local minima and two maxima. These positions in Figure 5a have been marked with arrows; these positions are used as the initial guesses for Gaussians n. 2, 3, 4, 5.

Once this choice has been made, the center of Gaussians 1 and 6 do not have a great impact on the final results since their contribution to the pulse shape is modest. Moreover, also the six widths and six amplitudes, as well as the offset, have a modest impact on the fitting results: this means that, even if these values were intentionally chosen far from the best ones, the algorithm could converge to an equally accurate solution. The six Gaussians obtained at the end of the fitting are reported in Figure 5b. The goodness of the fitting shown in comparing the measured and fitted reference pulse in Figure 5a is expresses by a low residue value, which is 8.4 × 10^−16^. The fitted reference pulse modeled according to Equation (1) has been used to determine layer thicknesses in the multi-layered sample. In order to understand the results obtained on more complex stratigraphy, such as the varnished areas in the multi-layered sample, several preliminary tests have been performed in the D areas—where there is no varnish layer.

**Figure 6:**
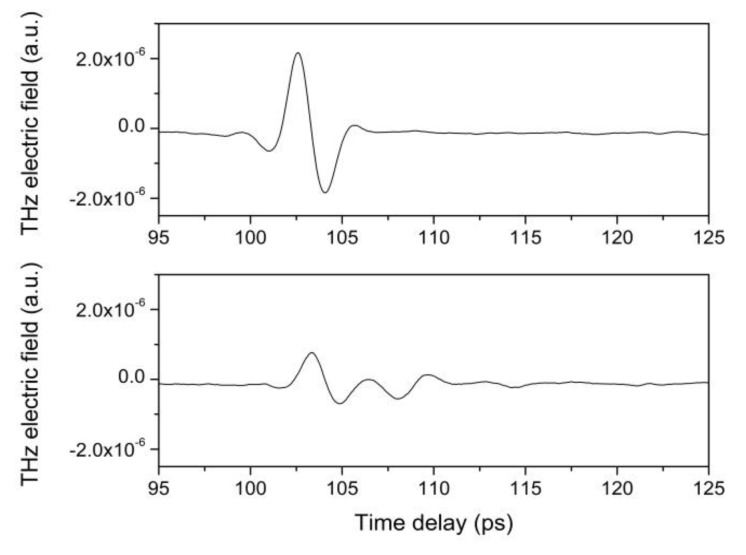
The upper image represents a THz pulse reflected from area D1 (metal on red bole layer) and the lower one, from area D2 (red bole on gypsum layer).

**Figure 7:**
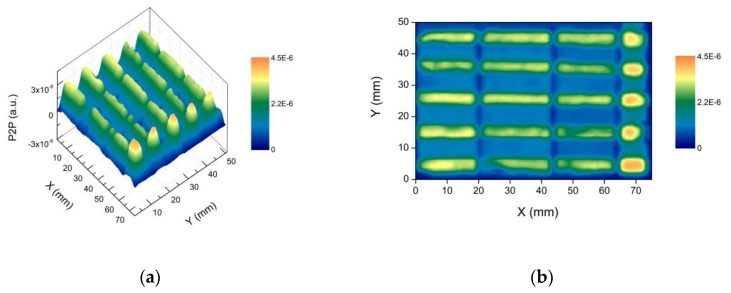
(**a**) THz imaging of the multi-layered sample: on the vertical axis is reported the difference between the maximum and the minimum of each waveform; on the horizontal plane, the measuring point coordinates; (**b**) the projection on the XY plane.

Two waveforms reflected from areas D1 and D2 are reported in Figure 6. It is interesting to note that, in the case of D1 (metal layer on red bole and gypsum layers) only one intense peak has been measured, since THz cannot penetrate metals, while in the case of D2 (red bole on gypsum layer), more than one peak can be observed. The temporal waveforms reflected from the entire sample have been recorded for each emitter-detector position with horizontal and vertical scan steps of 1 mm. This means that 3900 measurements have been performed. For each waveform, the difference between the maximum and minimum has been measured, and the results are reported in Figure 7.

This representation of the THz data makes it possible to locate the copper tape under the dielectric layers. In fact, the greatest difference can be associated with the reflection at metal interfaces.

Moreover, a decrease in this difference is visible where there is no metal layer or in transition between areas with different gypsum thicknesses (e.g., from A to B). The latter can be regarded as an edge effect, due to the finite dimension of the THz focused pulse (of the order of 1 mm at 1 THz), which is likely to partially overlap the two different stratigraphy.

**Figure 8:**
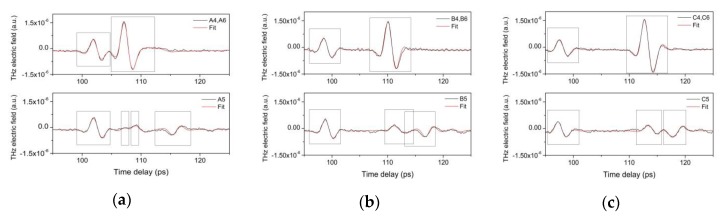
THz pulses reflected from different areas not covered by varnishes: (**a**) thin Gy1 on metal tape (upper image), thin Gy1 layer on red bole (lower image); (**b**) intermediate Gy1 on metal tape (upper image), intermediate Gy1 on red bole (lower image); (**c**) thick Gy1 on metal tape (upper image), thick Gy1 on red bole (lower image).

The fitting procedure has been first tested on the areas not covered with brass varnish. Six areas with a rather simple stratigraphy have been considered. The areas labeled with numbers 4 and 6 have gypsum, copper tape, red bole, and gypsum layers, and have been considered together; the areas labeled with the number 5 have the same stratigraphy as number 4, except for the copper tape that is absent. The different thicknesses of the outer gypsum layer (Gy1) characterize the areas labeled with the letters A, B, and C. They have been prepared with rather thin, intermediate and thick gypsum layers, respectively. For each area under test, the reflected signal from 51 different points has been considered. Six pulses representative of these areas are reported in Figure 8.

The first observation concerns the first peak position: a time delay in the first reflection has been evidenced in the three areas. It should be recalled that, in these experiments, a moving platform containing both the emitter and the detector has been used for imaging purposes. In this configuration, the sample is considered to be stationary, and the only moving element is the emitter-detector system. When the reflected signals from two different points evidence to a time delay in the first reflection, these two points do not have the same emitter-detector distance. In the present case, this is explained by considering that areas B and C have been prepared with thicker gypsum layers than those of area A.

Moreover, in the areas labeled with numbers 4 and 6, the second reflection has a more intense peak than in the areas labeled 5. In fact, the corresponding amplitudes (also obtained with the fitting procedure) are higher than 0.5. Moreover, it has been observed that no other reflections after the second one is present. These observations are justified by considering the presence of metal (copper tape) under the first gypsum layer. The boxes in Figure 8 have been introduced in order to highlight the reflected pulses.

Reflected pulses of 51 different measurement points in each area have been fitted in accordance with Equation (4). The results obtained in the case of layers not covered by any metal varnish, have been summarized in Table 1: the average values (in microns) and relative errors as the ratio between the standard deviation and the average have been reported. The thickness has been calculated using the time difference between two consecutive pulses, and the refractive index of each layer under test has been previously estimated using single-layer samples. In particular, for gypsum, and red bole layers, 2.04 and 2.55 have been obtained, respectively, for Shellac, Colophony, and Sandrac-based varnishes 1.86, 1.83, 1.88, respectively.

The thicknesses measured according to the proposed procedure can now be compared with those measured by analyzing cross-sections with optical microscope. The best agreement has been found with Gy1 and Gy2 layers; instead, in the case of thin layers (such as red bole), although there are differences in the results, the thicknesses measured with THz still overlap the measurement uncertainty intervals found by means of cross-section analysis. However, the agreement on the overall results can be considered as good: this enables us to regard the proposed procedure as being a valid non-destructive alternative to standard cross-section examination whenever the main aim of the latter is to measure thicknesses.

**Figure 9:**
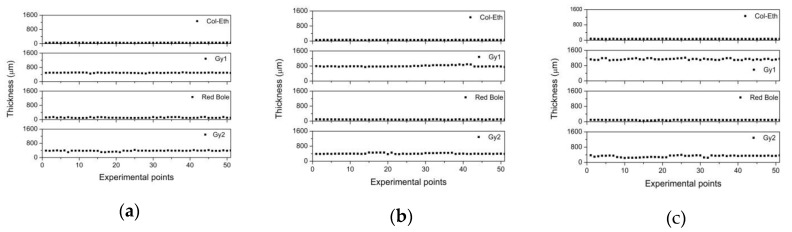
(**a**) Thicknesses of 51 points in areas A2; (**b**) area B2; (**c**) area C2. In the three cases the stratigraphy comprises varnish (Col-Eth), outer gypsum (Gy1), red bole (Red Bole) and inner gypsum (Gy2) layers.

The fitting procedure has been extended also to areas covered by varnish layers such as colophony-based varnish. The thickness measurements are presented in Figure 9: on the horizontal axis is reported the label of each of the 51 measured points considered, while on the vertical one the corresponding thickness is expressed in microns. It is interesting to note, that both the red bole and inner gypsum (Gy2) layers show quite uniform thicknesses in areas A2, B2, and C2. Instead, the most significant differences are in the outer gypsum layers (Gy1). These observations match the way in which the stratigraphy has been prepared.

The data presented in each graph of Figure 9 has been averaged, and the relative error has been calculated and reported in Table 2, for comparison with data obtained with the cross-section analysis (optical). A generally quite good overlap between THz and cross-section measurement uncertainty intervals has been found. 

This analysis has been performed also on the other two metal varnishes (Shellac and Sandrac-binders). The results summarized in Table 3 and Table 4 show a good agreement with those obtained through cross-section analysis (optical).

It should be noted, that in all the cases considered, the agreement has been considered to be good when there is an overlap (even partial) in the measurement uncertainty intervals. In general, the fitting procedure makes it possible for the first layer thickness to be accurately measured. In fact, it has been possible to estimate first layer thicknesses down to 40 microns. However, as the pulse penetrates into deeper layers, its peak-to-peak amplitude decreases. The reflected pulse can be equally fitted, but a shift in the temporal parameters may occur. This may be the responsible for the failure to overlap: these few cases are generally related to inner layers such as red bole on varnish and Gy1 layers.

The analysis method, preliminarily tested on the multi-layered prepared samples, has been eventually applied to the original fragment in order to characterize its stratigraphy. In particular, under the same experimental conditions as those of the multi-layered sample, the imaging of the entire fragment surface has been performed.

**Figure 10:**
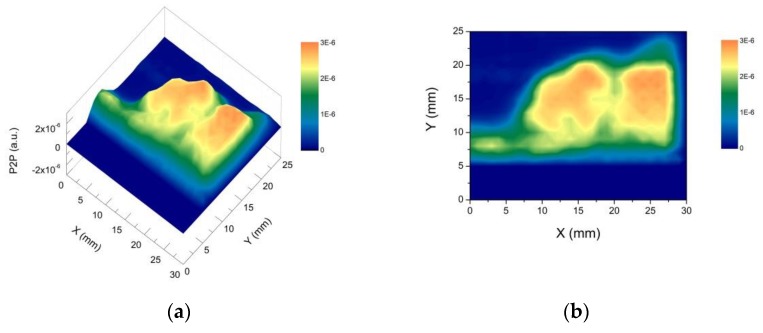
(**a**) THz imaging of the original fragment: on the vertical axis is reported the difference between the maximum and the minimum of each waveform; on the horizontal and vertical axes, the label of the measuring points; (**b**) THz horizontal projection; (**c**) color scale in arbitrary units.

The differences between the maximum and the minimum of each waveform are reported in Figure 10. The values in the central area resulted to be higher than in the peripheral one. The former is congruent with the presence of gold leaf relics.

**Figure 11:**
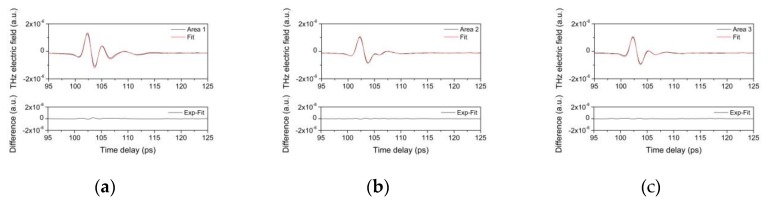
In the upper graphs are reported the THz pulse measured and the corresponding fit; in the lower graph, the differences between the measured and the fit data. (**a**) Reflection pulses from area 1; (**b**) area 2; (**c**) area 3, as schematized in Figure 1 (**a**).

In Figure 11, are reported pulses reflected from points located in three different areas into which the fragment can be divided, according to the schematization presented in Figure 1a, as well as the corresponding fits. The results are very accurate, as demonstrated by the difference between the experimental and fit data, which are also presented in Figure 11. Assuming 1.86 as refractive index for the varnish layer (corresponding to the average of the refractive index of Sandrac, Shellac and Colophony based varnishes); the fitting procedure makes it possible to estimate the layer thickness.

The results reported in Table 5 are consistent with those directly measured through the cross-section. It should be noted, that the THz pulse can penetrate to the red bole layer since there are micro-lacunas are in the gold leaf.

Moreover, since the fitting procedure, makes it possible to determine both the time delay and the amplitude of each reflected pulse, the lower amplitude values obtained for inner reflected pulses than the first pulses, determine that the gold leaf (metal layer) is not in a good state of conservation or completely lost in the measurement site of the areas 1. If there was a continuum metal layer, the detected amplitude of the reflected peak should have been greater than 0.5, as is obtained for example in the case of laboratory-prepared samples (Figure 2).

## 4. Conclusions

Thickness measurements of paint layers are generally accomplished in the frequency domain, and plenty of works in different applicative fields are known. Instead, in time domain, few studies are devoted to thickness measurements of metal paint layers in particular in automotive industries. In other applicative fields (such as in cultural heritage), the thickness measurements of metal paint layers that are not standardized as in automotive industries can be complicated by the extreme variability of the surfaces. In this work, a simplified model in time domain has been introduced in order to demonstrate the potential of THz reflectometry for characterizing overpaint layers on gilded surfaces, which were applied in restoration interventions of the past. The knowledge of the number and thicknesses of the paint layers applied upon an original gilded surface represents a frequent material knowledge problem in conservation of several artifacts of cultural interest. Here, we developed a non-invasive characterization approach using THz pulse-echo measurements and corresponding data elaboration based on Gaussian fits of the detected temporal profiles. The technique was calibrated through a suitable set of prepared samples and it was then applied for measuring the thicknesses of the typical material layers encountered in repainted picture frames of 19th century from the Rijksmuseum: gypsum re-preparation and brass paint. Thickness measurements were extended to the original bole layer through macro and micro-lacunas of the gold leaf in a similar way as THz radiation propagated through the interspaces of the metal particles of the brass paint.

In conclusion, the method presented can allow preliminary material characterization of originally gilded artifacts, which were patinated along the past, such as the present picture frames, sculptures, painting and other. This can be very useful in order to define suitable conservation treatments aimed at recovering the remnants of original gilding.

## Figures and Tables

**Table 1:** THz estimate and optical measurement of layer thickness (in microns) of six areas not covered by brass varnishes.

	A4, A6		A5		B4, B6		B5		C4, C6		C5	
	THz	Optical	THz	Optical	THz	Optical	THz	Optical	THz	Optical	THz	Optical
Gy1	426 ± 4%	424 ± 4%	425 ± 3%	424 ± 4%	798 ± 32%	828 ± 4%	798 ± 4%	828 ± 4%	1218 ± 3%	1196 ± 3%	1202 ± 4%	1196 ± 3
Red Bole	-	66 ± 23%	67 ± 9%	66 ± 23%	-	49 ± 28%	63 ± 5%	49 ± 28%	-	45 ± 40%	63 ± 39%	45 ± 40%
Gy2	-	469 ± 7%	499 ± 2%	469 ± 7%	-	370 ± 3%	350 ± 16%	370 ± 3%	-	366 ± 9%	342 ± 20%	366 ± 9%

**Table 2:** THz estimation and optical measurement of layer thickness (in microns) of six areas covered by Colophony-based varnish.

	A1, A3		A2		B1, B3		B2		C1, C3		C2	
	THz	Optical	THz	Optical	THz	Optical	THz	Optical	THz	Optical	THz	Optical
Col-Eth	54 ± 28%	40 ± 12%	55 ± 38%	40 ± 12%	70 ± 25%	51 ± 18%	60 ± 32%	51 ± 18%	69 ± 27%	45 ± 35%	67 ± 16%	45 ± 35%
Gy1	461 ± 19%	397 ± 7%	496 ± 9%	397 ± 7%	796 ± 9%	728 ± 6%	801 ± 12%	728 ± 6%	1129 ± 9%	1115 ± 5%	1138 ± 7%	1115 ± 5%
Red Bole	-	150 ± 27%	113 ± 35%	150 ± 27%	-	46 ± 9%	77 ± 16%	46 ± 9%	-	73 ± 23%	88 ± 38%	73 ± 23%
Gy2	-	384 ± 6%	379 ± 21%	384 ± 6%	-	380 ± 7%	408 ± 13%	380 ± 7%	-	372 ± 8%	325 ± 28%	372 ± 8%

**Table 3:** THz estimate and optical measurement of layer thickness (in microns) of six areas covered by Shellac-based varnish.

	A7		A8		B7		B8		C7		C8	
	THz	Optical	THz	Optical	THz	Optical	THz	Optical	THz	Optical	THz	Optical
She-Eth	60 ± 30%	52 ± 16%	62 ± 24%	52 ± 16%	53 ± 33%	46 ± 27%	55 ± 37%	46 ± 27%	62 ± 24%	75 ± 26%	67 ± 28%	75 ± 26%
Gy1	449 ± 26%	456 ± 8%	496 ± 32%	456 ± 8%	1033 ± 33%	920 ± 2%	1020 ± 12%	920 ± 2%	1141 ± 19%	1174 ± 4%	1126 ± 13%	1174 ± 4%
Red Bole	128 ± 31%	84 ± 21%	-	84 ± 21%	94 ± 17%	107 ± 34%	-	107 ± 34%	88 ± 18%	43 ± 30%	-	43 ± 30%
Gy2	570 ± 22%	498 ± 8%	-	498 ± 8%	287 ± 26%	293 ± 8%	-	293 ± 8%	440 ± 30%	368 ± 9%	-	368 ± 9%

**Table 4:** THz estimate and optical measurement of layer thickness (in microns) of six areas covered by Sandrac-based varnish.

	A10, A12		A11		B10, B12		B11		C10, C12		C11	
	THz	Optical	THz	Optical	THz	Optical	THz	Optical	THz	Optical	THz	Optical
Oil-San	59 ± 32%	59 ± 26%	69 ± 16%	59 ± 26%	130 ± 39%	105 ± 24%	67 ± 37%	105 ± 24%	60 ± 22%	89 ± 8%	70 ± 15%	89 ± 8%
Gy1	553 ± 23%	516 ± 8%	574 ± 25%	516 ± 8%	628 ± 25%	689 ± 5%	657 ± 21%	689 ± 5%	1152 ± 18%	1102 ± 8%	1161 ± 6%	1102 ± 8%
Red Bole	82 ± 20%	86 ± 13%	--	86 ± 13%	126 ± 32%	89 ± 30%	--	89 ± 30%	89 ± 17%	58 ± 20%	--	58 ± 20%
Gy2	491 ± 39%	460 ± 6%	--	460 ± 6%	439 ± 32%	405 ± 15%	--	405 ± 15%	504 ± 12%	436 ± 6%	--	436 ± 6%

**Table 5:** Thickness (in microns) of layers in the three points of the original fragment (see Figure 1 and Figure 11).

	1	2	3
Varnish	33	-	-
Gy1	180	160	-
Red Bole	66	46	66
